# Supplementary material for: On closed-form tight bounds and approximations for the median of a gamma distribution
Source: PLoS One. 2021 May 13;16(5):e0251626. doi: 10.1371/journal.pone.0251626 (PMC8118309; doi:10.1371/journal.pone.0251626)
Supplement: Attachment — Submitted filename: PLOS ONE review 2021.pdf [file pone.0251626.s001.pdf]

Review of "Closed-form tight bounds and approximations for the median of a gamma distribution" by Richard F. Lyon

Summary and recommendation

Since the work of Chen and Rubin in 1986 (reference 1), the problem of obtaining accurate bounds and approximations for the median of the gamma distribution has received much interest. The interest here arises because the median is one of the most basic quantities in probability and statistics, but, for general parameter values, no closed-form formula is available for the median of the gamma distribution (a widely used probability distribution).

This paper contributes to this problem. The contributions are the following. (i) A tight, rigorously proven lower bound for the median, given on page 3. At least to me this seems new, although the author should clarify this (see a below specific comment). (ii) Asymptotic approximations for the median. (iii) **Conjectured** lower and upper bounds for the median that are in some sense best possible and improve on some results from the existing literature (although see a below comment, which the author should address). These conjectured bounds are supported by non-rigorous, but convincing arguments based on asymptotic and graphical considerations, and are in all likelihood true bounds. (iv) Interpolation formulas between the conjectured lower and upper bounds that give accurate approximations for the median.

Other than the simple lower bound on page 3 (which may be new), the lower and upper bounds are not rigorously derived. In this sense, from a mathematician's point of view, this paper lacks hard results, and a critical mathematician would view this work as the early stage findings of an unfinished research project. However, I consider this to be a worthwhile paper on an interesting topic and am prepared to make a positive recommendation. Let me explain why.

Firstly, the problem of estimating the median of the gamma distribution is one that can be understood by a broad scientific audience, and the results (upper and lower bounds and approximations for the median) can be appreciated and understood by a broad scientific audience. In addition, the problem has historical interest. The contribution of accurate conjectured lower and upper bounds for the median is worthwhile. Based on results from a paper of Berg and Pedersen (reference 2), the author has sought to find upper and lower bounds of the form $2^{-1/k}(A+Bk)$, where $k > 0$ is the rate parameter of the gamma distribution. Some intelligent experimenting based on asymptotic analysis then allows the author to (non-rigorously) find values of A and B that are best possible, allowing the author to improve on existing bounds of Berg and Pedersen. Whilst the work is non-rigorous, the combination of the explanations given by the author and the graphical and numerical results does convince the reader that these bounds are in all likelihood true bounds that are best possible for the family of bounds considered by the author. Because the reader can be confident the bounds are true, this paper may stimulate further research from mathematicians who wish to rigorously prove the bounds. The author then, for I think the first time in the literature, and certainly the first time in this level of detail, presents interpolating formulas for the median of the gamma distribution based on these bounds, which lead to estimates for the median, which through numerical experiments are seen to be very accurate. These results could

be useful for researchers wishing to practically find a quick and accurate estimate for the median of the gamma distribution.

In summary, I consider this paper to be a worthwhile contribution to an interesting problem that is of broad interest. The paper may stimulate further research from mathematicians to verify the author's conjectured findings. The numerical analysis supporting the conjectured bounds is performed to a high technical standard with an appropriate level of detail. I found the paper to be clear and easy to follow. PLOS ONE seems a reasonable fit to me. Provided the author seriously addresses the below general and specific comments, I would support the publication of this paper in PLOS ONE.

General comments

(1) Throughout this report, I have been careful to stress that most of the author's bounds are conjectured. This is something the author needs to do more clearly. I acknowledge that the author does put an asterisk to denote bounds that have not been rigorously proved and writes in the conclusion "Proving that they [the conjectured bounds] are in fact bounds remains an unmet challenge." However, the point that the bounds are conjectured needs to be made very clear early in the paper. Indeed, it wasn't until I reached page 4 of this short paper that I realised that the bounds were not going to be rigorously proven, which is what one expects when reading a paper on mathematics. It is important that the reader is made aware at an early stage that the bounds are conjectured. For example, if another researcher uses the author's bounds to bound a quantity themselves then the bound they obtain is in turn non-rigorous, and they must be made aware of this.

The author therefore needs to make the following edits. The title must change. An example would be "Approximations and conjectured closed-form tight bounds for the median of a gamma distribution", or if the author prefers not to use the word 'conjecture' in the title, something like "On closed-form tight bounds and approximations for the median of a gamma distribution". The author must clearly state in the Abstract that the bounds are conjectured, and can mention that they are supported by asymptotic, numerical and graphical arguments. It must be mentioned in the Introduction that the bounds are conjectured. The first sentence of the Conclusion should be edited from "Tight upper and lower bounds to the median of the gamma distribution are introduced" to something like "Conjectured tight upper and lower bounds to the median of the gamma distribution are introduced".

Moreover, the author may wish to modify certain sentences throughout the paper to reflect the more modest findings of this research. For example, the sentence on page 5 that ends with "this seems reliable enough in concluding that these are bounds (but mathematicians are invited to interpret these as conjectures to be proved)" needs to be edited. There is no doubt that the bounds are conjectures to be proved.

(2) On page 2, the author presents the well-known two-sided inequality of Chen and Rubin for the true mean ν of a gamma distribution with rate parameter ν and scale parameter 1: $k-1/3 < \nu < k$. Throughout the paper the author

compares his bounds to these bounds. However, for $k \geq 1$, the upper bound can be significantly improved: Gaunt and Merkle [1, Theorem 3.1] give the upper bound

$$\nu < k - 1 + \log(2), \quad k > 1;$$

note that there is equality at $k = 1$, which corresponds to the case of the exponential distribution. This upper bound extends the range of validity of a previous bound of Choi (reference 3) from positive integer k to all $k > 1$. As $1 - \log(2) \approx 0.306852$ is 'close' to $1/3$, when this upper bound is combined with the lower bound $k - 1/3$ it results in a very accurate two-sided inequality for the median.

The author should report this bound of [1] in either the 'Problem formulation' or 'Prior work' sections. The author should also update their numerical results that give comparisons to existing bounds to include a comparison to this bound. As this upper bound is very accurate, some of the author's conclusions regarding the performance of his bounds relative to others in the literature may change.

The paper [1] (see Section 3) also gives conjectured inequalities for the median of the variance-gamma distribution. In particular, the authors conjecture that the median of the variance-gamma distribution can be bounded above and below by the median of certain gamma distributions. Therefore the author's conjectured accurate bounds may in turn, at least for some parameter values, result in improved conjectured bounds for the median of variance-gamma distribution. I will leave up to the author to decide whether they wish to add such a remark to their paper.

Specific comments

(1) Page 1, Abstract (and elsewhere): The author uses "closed-form" and closed form. Please consistently use just one of these.

(2) Page 1, Introduction: I suggest the author brings Tables 1 and 2 to the reader's attention in the introduction. These tables are very useful and efficiently display the main findings of this work. A reader who wants to quickly get to the results would find such a comment in the introduction to be very helpful.

(3) Page 1, At the bottom of the page, the author introduces the notation ν for the median of the gamma distribution with rate parameter 1 and scale parameter k. I suggest modifying this notation to $\nu(k)$ to emphasis the dependence on k. Later in the paper, the author sometimes does write $\nu(k)$, and it would be helpful to keep things consistent throughout the paper. The same comment applies to the author's notation for other bounds and approximations: e.g., ν_{L_∞} becomes $\nu_{L_\infty}(k)$.

(4) Page 3: The author neatly derives the bound $\nu > 2^{-1/k}\Gamma(k+1)^{1/k}$. I appreciate that the author derives this bound with the intention of motivating further work in the paper. Nevertheless, it would be helpful if the author were to give a statement as to whether they believe this to be a new result.

(5) Page 4, line -3: $\log(x)$ is not defined at $x = 0$, so it makes no sense to write $\log(0)$. The author therefore may consider formulating "$\log(0)$ cusps" differently.

(6) Page 5, line -9, "The slope $v(k)$": Does the author mean $\nu(k)$? Please look out for other such typos, e.g., in the displayed equation below. Also, it would be helpful to the reader if the author were to provide an explanation or short calculation to verify how they found the formula for $v'(k)|_{k=1}$.

(7) Page 8: In the final displayed equation, the comma should be a full stop.

References

[1] Gaunt, R. E. and Merkle, M. On bounds for the mode and median of the generalized hyperbolic and related distributions. *Journal of Mathematical Analysis and Applications* **493** (2021), Article 124508.
