# Supplementary material for: On closed-form tight bounds and approximations for the median of a gamma distribution
Source: PLoS One. 2021 May 13;16(5):e0251626. doi: 10.1371/journal.pone.0251626 (PMC8118309; doi:10.1371/journal.pone.0251626)
Supplement: Attachment — Submitted filename: PLOS ONE review rev1 2021.pdf [file pone.0251626.s003.pdf]

Review of the revision "On closed-form tight bounds and approximations for the median of a gamma distribution" by Richard F. Lyon

Summary and recommendation

The author has carefully addressed my comments and I am happy with the revision. I recommend that this paper is now ready for publication in PLOS ONE.

Specific comments

(1) P. 2, Prior work section: It would be more standard to use the notation $k \geq 1$ rather than $k >= 1$.

(2) P. 2: In the final two displayed approximations for the median, the notation ν is given, rather than $\nu(k)$. It would be good to keep the notation consistent.
