# Supplementary material for: On closed-form tight bounds and approximations for the median of a gamma distribution
Source: PLoS One. 2021 May 13;16(5):e0251626. doi: 10.1371/journal.pone.0251626 (PMC8118309; doi:10.1371/journal.pone.0251626)
Supplement: Attachment — Submitted filename: Response_to_Reviewers.pdf [file pone.0251626.s004.pdf]

Rebuttal Letter / Response to Reviewers

PONE-D-21-00917R1
On closed-form tight bounds and approximations for the median of a gamma distribution
PLOS ONE
Richard F. Lyon, author

I thank the reviewers again, and the editor, for the constructive suggestions and positive reaction to my article. I have incorporated the latest suggestions, pretty nearly, and a few minor corrections.

Editor suggests a rewrite of the Abstract. I did that, starting with his suggested opening but then with some changes. Instead of "with a fixed shape parameter k" I said "as a function of shape parameter k". I moved the algebraic form of the bounds up one sentence to near the bounds it most particularly applied to. And I added a few more words to clarify things.

Editor suggests dropping the short Introduction paragraph and merging the next two sections into an Introduction. Done; exactly as suggested, except also moved the sentence saying that results are summarized in tables to come, since the reviewers had asked for that.

Editor says an added proof that "$\Gamma(k + 1)^{1/k}$ increases monotonically from $e^{-\gamma}$, ..." would be welcome, so I added that.

Reviewer 1 pointed out notation error in >= relation. I found and fixed 2 of those.

Reviewer 1 pointed out that I failed to add the "(k)" in a few places. I found and fixed 9 of those (including 5 in table 2).

Reviewer 2 had no specific comments.

Other things I changed:

Fig 2 had an error in the y axis label, so I fixed that.

I modified the comment on the potential use of quantile mechanics partial sums as bounds, as they are not closed form as I had suggested.

I re-ordered references to match the change order of citation in the new introduction.

I added an acknowledgement name, for the colleague who helped me with the proof.

That's all.
